# “On papers”: perceptions of synthetic cannabinoid use among black males under criminal justice supervision

**DOI:** 10.1186/s40352-016-0032-z

**Published:** 2016-01-27

**Authors:** Joseph B. Richardson, Christopher St. Vil, Eric Wish, Carnell Cooper

**Affiliations:** 1 Department of African-American Studies, University of Maryland at College Park, 1119 Taliaferro Hall, College Park, MD 20742 USA; 2 Center for Substance Abuse Research (CESAR), University of Maryland, College Park, 4321 Hartwick Road, Suite 501, College Park, MD 20740 USA; 3 School of Medicine, University of Maryland Medical Center, 22 South Greene Street, Baltimore, MD 21201 USA; 4 School of Social Work, University at Buffalo, 685 Baldy Hall, Buffalo, NY 14260 USA

## Abstract

**Background:**

A number of studies reveal a strong linkage between SC use and avoiding positiveurine creens. Despite this work and given the high rates of criminal justice supervision among Black men in the U.S., little is known about SC usage among Black men under criminal justice supervision.

**Methods:**

In-depth qualitative interviews were conducted with 11 Black men under criminal justicesupervision treated by an urban ED for violent injury.

**Results:**

Themes that emerged from the analysis include 1) prevalence of use, 2)health literacy, 3) availability and costs, 4) negative side effects, and 5) criminal justice supervision.

**Conclusions:**

Criminal justice supervision policies are a contributing factor to SC use among Black men under criminal justice supervision.

*This was never intended to be used in people… It even says on the label, ‘Not for human consumption.’ Ironically, that’s the only accurate thing on the label. This is not marijuana. It should not be thought of like marijuana. We have to get this out there: Its effects are serious. It’s a totally different drug. (Walton* 2014 *)*

## Background

Reports on the use of synthetic cannabinoid (SC) use among adolescents and young adults in the U.S. differ and present a conflicting picture depending on the source of the report. According to the Monitoring the Future Survey (MTF), in 2012 SC ranked second to marijuana among illicit drugs used in the past year by high school seniors with 11.4 % reporting use. In the 2 years since the survey has been tracking SC use, 5.8 % of 12^th^ graders reported its use in 2014, compared with 7.9 % in 2013 representing a cumulative 5 % decrease. This decrease was associated with an increase in the perceived risk of taking SC once or twice among 12^th^ graders even though the level of the perceived risk was reported as low (Johnston et al. 2014). Despite this decline in SC usage reported by the MTF, there has been an alarming rise among both teens and young adults presenting to emergency departments (ED) for reasons involving SC fueling public health concerns (Harris and Brown 2013).

The American Association of Poison Control Centers (2012) reported that the number of toxic reactions to SC has increased drastically. In 2010, Poison Control Centers (PCC) received approximately 2900 calls reporting adverse effects from SC. By 2011, PCCs reported 7000 calls. According to data available from the Drug Abuse Warning Network (DAWN) an estimated 11,406 U.S. emergency department (ED) visits in 2010 involved a SC product, and three-fourths of these visits were made by patients 12–29 years of age. In the majority (59 %) of ED visits made by patients’ ages 12–29 that involved SC, no other substances were involved. SC were used in combination with one other substance (i.e., marijuana, pharmaceuticals, and alcohol) in 36 % of the visits. By 2011, there were an estimated 28, 531 ED visits involving a SC product (57 % were SC alone), more than double the rate reported the previous year. Furthermore, despite the classification of SC in schedule I under the Controlled Substance Act, SC products continue to evolve due to modified chemical structures and continue to be marketed widely. A report released by the Center for Behavioral Health Statistics and Quality (Substance Abuse and Mental Health Services Administration and Center for Behavioral Health Statistics and Quality 2014) suggested that the chemicals identified in SC products confiscated and identified during the years 2010 and 2013 are vastly different. Additionally, the availability of SC has surged as indicated by the rise of laboratory reports. In 2010 there were 469 laboratory reports between January and June. In 2013, between those same months, there were 17, 241 laboratory reports associated with SC usage.

SC constitute a significant public health concern because they mimic natural marijuana-like effects, are readily available, and undetectable by conventional drug-screening tests which makes the substance particularly appealing to young people (Fattore and Fratta 2011). Although data remains limited on the induction and duration of adverse effects of SC on humans, the most common reported adverse clinical effects include psychosis (Every-Palmer 2010; 2011), seizures (Lapoint et al 2011), agitation (Forrester et al. 2011), tachycardia (Simmons et al. 2011), and cardiotoxicity (Forrester et al. 2011). Reflective of this ongoing public health concern with SC are the reports of adverse effects by public health officials in different US states. In January the University of Massachusetts, Division of Medical Toxicology (2015) submitted a report to the National Institute of Drug Abuse National Early Warning System Network titled “Synthetic Cannabinoid Use Case Summaries: Initial Report on Resurgence.” This report highlighted case studies of high school students who presented to EDs following SC use in school. Similarly, a New York Times article (Schwarz 2015) reported that state poison control centers received about 1000 reports of adverse reactions to SC in the first three weeks of April more than doubling the total from January through March. A Washington Post article (Hauslohner and Hermann 2015) in July citing data from the Washington, DC Police, Fire, and EMS departments reported that since August 2012, SC cases remained below thirty a month until May 2014 when they began seeing at least 50 cases a month. In June of 2015 that figure spiked to 439 patients. A more recent report from the New York City Department of Health and Hygiene (2015) documented more than 2300 SC-related emergency department visits during the months of July and August in 2015. While MTF survey data gives reason for optimism, the testimonies from DC, New York, and Massachusetts along with DAWN data call into question the conclusion put forth by MTF and validate the sustained public health concern with SC.

## Previous research

Research around SC has largely been associated with two areas. The first area of research is concerned with the toxicology and pharmacology of SC (Seely et al. 2012). This strand of research seeks to identify the chemical composition SC products and examine the biological, physiological and neurological effects of SC consumption. The second strand of research seeks to establish user profiles and identify the motivations for SC use among different populations and whether the predictors of use differ by various characteristics (Perrone et al. 2012; Vandrey et al. 2012; Walker et al. 2014). Taken together, this research has increased our understanding of who engages in SC use and why.

Primary users tend to be adolescent and young adult males, who smoke marijuana, and are seeking to avoid legal complications (Seely et al. 2012). The popularity of SC is rooted in not being easily detected in typical urine drug screenings for THC (Forrester et al. 2012). As a result, those most likely to engage in SC use are males who engage in regular marijuana use and seek to participate in activities where random or initial drug screens are anticipated (i.e., military, sports teams, employment) or a condition of compliance (criminal justice supervision). Reasons for use cited in previous research include being under criminal justice supervision (Perrone et al. 2012; Vandrey et al. 2012), seeking employment (Perrone et al. 2012), residing in a sober living facility (Perrone, et al. 2012), or joining or being an active member of the military (Perrone et al. 2012; Walker et al. 2014; Bebarta et al. 2012; Johnson et al. 2011). Although, research on SC has improved our understanding of who is most likely to use and why, more research needs to be conducted on the prevalence of SC use across diverse populations and settings. The Vandrey et al. (2012) study was an internet based survey with an overwhelmingly white male sample. The Perrone et al. (2012) study used a primarily Hispanic undergraduate sample for the quantitative portion of the study and white heterosexual males for the qualitative portion, and the Walker et al. (2014) study focused on active duty personnel. This study is an addition to the literature around SC use by interviewing Black males under criminal justice supervision about the prevalence of use among themselves and in their communities.

## Avoiding dirty urine: legal consequences of criminal justice supervision

One explanation that may increase the likelihood of SC use among Black men is disproportionate criminal justice involvement. According to the most recent data from the Bureau of Justice Statistics (Carson 2014) non-Hispanic Blacks comprise the largest portion of male inmates under state or federal jurisdiction in 2013, compared to non-Hispanic Whites and Hispanics. Almost 3 % of Black male U.S. residents of all ages were imprisoned on December 31, 2013, compared to 0.5 % of White males. The higher, disproportionate rate of imprisonment for Black males translate into higher rates of criminal justice supervision (i.e., probation or parole). The BJS definition for criminal justice supervision includes adults on probation, parole, or any other post-prison supervision (Herberman and Bonczar 2015). The probation and parole rates for Blacks nationwide are 2.9 and 5.2 times those for Whites respectively even though Whites make up 70 % of all the arrests in the U.S. (Hartney and Vuong 2009). Since studies of SC use have found that individuals under criminal justice supervision are more likely to use SC (Harris and Brown 2013; Perrone et al. 2012; Vandrey et al. 2012) it is reasonable to assume that the disproportionate rate of incarceration and criminal justice supervision among Black men may increase their likelihood for SC use. The purpose of this study is to explore the prevalence of SC use among black males who experience criminal justice supervision and their motivations for use.

## Methods

The sample for this study was extracted from a larger sample of a primary research project. In the following section we first describe the recruitment procedures for the primary study, then describe how the sub-sample was derived for the present study.

## Recruitment for the primary study

The primary study was conducted at an urban hospital center in Prince George’s County, MD, a predominately Black county of almost 900,00 residents that borders the District of Columbia. The overall sample for this study was recruited for a longitudinal ethnographic pilot study examining risk factors for recurrent violent injury, linkages, and barriers to care, as well as HIV risk behaviors among young Black men who have been violently victimized (i.e., gunshot wound, stabbing or assault) and treated at the Prince George’s County Hospital Trauma Center. Patients were initially screened for eligibility using daily CERNER logs (computer generated data on each individual patient) generated by the hospital’s trauma unit. Inclusion criteria for the study were: self-identifying as male and black, between the ages of 18–34, experiencing a violent injury (gunshot wound, stabbing, or assault), and being treated by the trauma unit. Exclusion criteria consistent with previous research conducted on victims of violent injury (Rich and Grey 2005) included: 1) being under police supervision/police custody, 2) experiencing traumatic brain injury or a severe head injury, 3) experiencing a self-inflicted injury that may impede the patient’s ability to understand the purpose of the study and provide informed consent, 4) not identifying as male, and 5) having a history of severe psychiatric disorder. An affirmative response to any of these exclusionary conditions rendered a potential participant ineligible for the primary study. Overall, 116 patients who met the criteria for eligibility were approached for participation in the primary study of which 24 (21 %) consented to participate. The sample had a median age of 22 years and a mean of 24.9 years.

## Procedures

Determination and protocol of who will be selected for inclusion to the study begins with an examination of the CERNER daily admissions roster followed by informal conversations with the charge nurse on the trauma unit. A cursory review of the CERNER daily admissions roster by VIP program staff provides a brief profile of each patient on the floor including the patients name, age, gender, type of injury, location of injury, date of birth, date of admission, and anticipated date/time of discharge. Patients who are determined from the CERNER daily admissions roster to identify their gender other than male (i.e., transgendered male to female), or who report their age as under 18 or over 34 are rendered ineligible to receive an eligibility assessment. In this way, the floor log serves as a sort of preliminary screener of patients that may be included or excluded from solicitation into the proposed study.

Other information crucial to determining whether or not a patient would be approached for an eligibility assessment was gathered by informal conversations with the charge nurses on the trauma floor. The charge nurse is the supervisor for each shift and they are privy to information that may not be located on the CERNER log but may assist members of the research team in determining whether a patient should receive an eligibility assessment. For instance, information about the patient being in law enforcement custody will not be available through the CERNER. In this case, the charge nurse will inform the researchers of that information and prevent them from approaching a patient who would ineligible for participation in the study even though they meet the inclusion criteria. Furthermore, if the charge nurse does not believe the patient is able to provide informed consent, we will use their judgment as the final determination on whether a patient gets approached for recruitment.

Patients fitting the inclusion criteria based on discussions with the charge nurse and an examination of the CERNER log were approached for recruitment by a member of the research team at bedside and administered an eligibility assessment. Those who were determined to be eligible received further information about the study and were asked to participate. We used in-depth semi-structured life history interviews at three time points (1 week after discharge from the hospital; 3 months; and 12 months). The interview guide covered a range of domains: previous hospitalizations, substance abuse, social support, previous and current involvement with the criminal justice system, neighborhood perceptions, exposures to violence, health insurance coverage and access to care, masculinity, social media, and HIV risk behaviors. Basic demographic information was also gathered, including insurance status, marital status, and employment history. Participants were compensated $50.00 for each completed interview.

## Analysis and coding for the present study

Twenty-four [*N* = 24] interviews were audio recorded and transcribed. Two principal investigators and two research assistants (unfamiliar with the data) independently coded each of the 24 transcripts to note major themes, concepts, and patterns associated with substance use and SC (Richardson and Robillard 2013). Coding decisions were compared and reflected a high level of consistency. This procedure allowed the coders to conduct reliability checks to ensure consistency with regard to the interview data collection. The data were coded and analyzed using Dedoose qualitative data analysis software. A Grounded Theory approach was used to generate emerging themes from the data (Glaser and Strauss 1967).

## Sub-sample demographics

Of the 24 study participants, 11 (46 %) reported on the prevalence of SC use in their communities. This analyses focuses on the responses from those 11 participants. The data used for the analysis was drawn from the first wave of interviews. A total of 11 interviews were transcribed, analyzed and coded.

Table 1. Presents the demographic characteristics of the study participants. Four (36 %) of the respondents received their HS diploma and one reported obtaining their GED. Five (45 %) of the respondents did not complete the 11^th^ grade and four (36 %) only completed compulsory schooling up to the 10^th^ grade. Five (45 %) of the respondents reported being employed and 10 (91 %) of the respondents reported some level of criminal justice involvement either in the form of probation or parole. Seven respondents in the sub-sample (64 %) reported engaging in marijuana use and nine (82 %) reported drinking alcohol. Prevalence of SC use was categorized as present or past use.

**Table 1:** Characteristics of analytical sample (*N* = 11)

Name	Age	Highest grade level completed	Employment Status	Criminal justice Invol.	Marijuana	Alcohol	Synthetic Cannabinoids	
							Present use	Past use
Mike J.	20	H.S. Diploma	Un-employed	No	X	X	X	
Peyton	21	H.S. Diploma	Employed	Yes		X		
Aaron	24	H.S. Diploma	Employed	Yes	X			X
Dan	22	10^th^ grade	Un-employed	Yes	X	X		X
Drew	33	H.S. Diploma	Employed	Yes	X	X		
Russell	21	9^th^ grade	Un-employed	Yes	X	X		X
Warren	19	11^th^ grade	Un-employed	Yes	X	X		X
Vinnie	19	9^th^ grade	Employed	Yes	X	X		
Kyrie	29	H.S. Diploma	Un-employed	Yes		X		X
Eli	32	10^th^ grade/GED	Un-employed	Yes				
Eric	34	H.S. Diploma	Employed	Yes		X		

## Results

Five themes emerged from the data analysis: 1) prevalence of use, 2) health literacy (which we defined as knowledge of the chemical compounds in SC, 3) availability and costs, 4) negative side effects and, 5) On papers: Criminal justice supervision.

## Prevalence of use

When asked about the prevalence of SC use in their communities knowledge of use stemmed from individual use of SC or observing use among their peers and/or others in their communities. Here Peyton describes the prevalence of use in his neighborhood.

*At one point though, I could say that, everyone I knew was off [smoking] that Scooby Snax stuff [brand of SC].*

Dan reported that the prevalence of use in his community was being driven primarily by the disproportionate number of young Black men in his neighborhood who were under some form of criminal justice supervision.

You got so many youngins [youth and young adults] that’s on papers [probation] they like f**k it, why not smoke it.

Warren also describes how the prevalence of SC use is correlated to the disproportionate number of community residents under community supervised corrections.

*A lot of people smoke it, because everybody is on papers [probation].*

The quotes presented here demonstrate how the study participants perceive the prevalence of SC use in their communities. The terms used by the participants such as, “everyone I knew” (Peyton), “so many youngins” (Dan), and “a lot of people…because everybody” (Warren) reflect the perception that a significant number of individuals in their communities and their networks are using SC. That the participants are connecting the high volume of SC use in their community to those who are “on papers” suggests not only that some of the use in the communities of these young men can be partly attributed to being under criminal justice supervision, but that there are a significant number of individuals in the these communities that are experiencing criminal justice supervision.

## Health literacy/ knowledge of chemical compounds in SC

Four participants (36 %) in the sub-sample reported a lack of knowledge regarding the chemical composition of SC. These participants acknowledged that they had no knowledge of the chemical compounds they were smoking. Dan reported:

I’ve seen people get addicted to it, but nobody knows what’s in that s**t. They (companies that produce synthetic marijuana) adding extra chemicals in that s**t. There’s something wrong with that s**t, especially with the police raiding stores and s**t.

Another respondent, Eli, compared smoking SC to PCP:

*They say that it’s not for human consumption. If I smoke that I might as well smoke some PCP.*

We found Eli’s comments insightful because some of the acute clinical effects of SC abuse mimic the effects of PCP such as aggression, irritability, hallucinations, paranoia and psychosis. Dan reported that smoking SC provided a high similar to PCP:

*I smoke weed when I want to mellow out, but when I want to get high, I mean real high, I need that Bizarro (brand of SC). It [SC]gives me a boat [street term used for PCP] high.*

One of the respondents Aaron described a bad experience after smoking SC and self-reflected on what was in it:

I said to myself, I don’t know why I’m doing this. I was ass naked and I just kept walking around. I said this thing is laced with s**t. When I laid down, I’m like, I’m never smoking that s**t ever again. I don’t know what they put in that s**t, that s**t is not right.

The quotes under the health literacy theme reflect the study participants’ perception that the ingredients in SC products being purchased by themselves or their peers are unknown and harmful. There is a perception by the study participants that SC contains extra chemicals that lead to erratic behavior such as in the case of Aaron and while it is clearly labeled on SC products “not for human consumption,” there are those who engage its use anyway. Additionally, study participants have compared the effects of SC to PCP, rather than natural marijuana, hence, providing an alternative to the assumption that SC produces cannabis-like effects.

## Availability and costs

Five participants (45 %) discussed the availability and relatively low cost of SC compared to natural-marijuana. Here Aaron reported that store owners actively market the drug to store customers:

People tried to sell it to me yesterday at the gas station… I mean the gas store station was trying to sell it to me. They were actually pushing that s**t. Like they can’t get it out of their store, they asking for people to buy it, like, we got bizarro for sale.

Despite legislation in Prince George’s County (research study site) prohibiting the sale of SC, respondents commented that many stores continue to sell it. Peyton discusses how stores continue to sell SC despite its illegality:

*I seen the news and they saying it’s illegal but these stores still selling that stuff to people. I didn’t know it was illegal, cause, it’s so easy to get it, you can walk into a corner store and ask ‘You got Scooby Snax’ [popular brand of SC]. They go and get it for you.*

One participant, Dan reported that several individuals in his neighborhood were producing their own SC, packaging it and selling it:

*Some people are making it [SC] at their crib [home] and selling it on the street. You know when it’s homemade because the package don’t have a barcode on it.*

Participants also reported that the low cost of the SC significantly increases use among youth and young adults in their neighborhoods. Participants compared the relative low cost of SC to high-grade marijuana. Here Dan describes the number of blunts he smokes of SC compared to high-grade marijuana and the price point:

*Man, for ten dollars I was getting one blunt or two blunts [high-grade marijuana]. I get ten blunts from that Scooby [brand of SC]. And I’m getting real high!*

Russell elaborates on getting more for his money when purchasing SC:

My folks would probably give me an ounce of that s**t, some K2 (brand of SC) for 20–$30 dollars Seven grams or a quarter ounce of real weed on the street is about 90–$120 dollars.

Warren describes the number of blunts he smokes from a $10 package of SC to a $10 (dime bag) of regular marijuana:

*I probably like 8 blunts from a bag of Scooby and like 2 [blunts] from a dime of reggie [regular marijuana].*

While previous research has indicated the availability of SC in vendor outlets, the participants of this study suggest a much more aggressive marketing campaign taking place in their communities. Availability of SC is rampant given the sheer volume of vendors within the community who are selling it and according to the study participants, some vendors continue to sell it despite its prohibition. Additionally, the significantly cheaper cost of SC in comparison to natural-marijuana makes it a more attractive alternative.

## Negative side effects

Eight participants (73 %) reported on the negative side effects of SC use. Some participants commented on the negative side effects they observed among peers and community residents while others reported on the negative side effects they experienced as users of SC products themselves.

Mike J, discusses how he has observed individuals smoking SC inappropriately. From his perspective as a user and observer he elaborates on how some users cannot regulate their use:

*They smoke the whole J [blunt] in one minute. They don’t put it out or nothing. That’s how they be ruining their brains and what not…going out of control…I don’t know…I never had no bad experiences with it [SC]… They don’t know how to smoke that. You gotta pace yourself. They think its regular weed.*

Peyton describes the negative side effects he observed among peers who use SC:

*Cause I know a lot of people who smoke the finest weed but they smoke that K2, they act retarded and their stomach hurt, or their going to throw up, or it makes them go crazy, Yeah, it’s going crazy man. It’s something I don’t ever want to witness people doing. I would show you a clip on my phone of my friends and how they acting…they be smoking that stuff they can’t stop moving, it made my friend pass out. My other friend, every time he smoke it he thinks he’s a rapper and starts performing, he get up and performs and dances and stuff. We be thinking he be playing but he does it over, and over, and over.*

Participants also discussed how older men in the community provide neighborhood youth with packs of SC in order to get them high enough to engage in violence. Once intoxicated, older men order youth to carry out ‘hits’ or violence against other neighborhood men. Warren describes this process:

*You can get them [adolescents] to do anything, you can get them to ride through the neighborhood and crush somebody [kill]. They [older neighborhood men] can get them [adolescents] to do whatever for these Scooby Snax, man.*

Here Dan reflects on an incident where he experienced the negative side effects of SC after smoking SC daily for an entire week. Dan describes experiencing hallucinations, paranoia and aggressive behavior:

So I just stripped down and started running, then I jumped up on a traffic light and hung there naked until the cops came. I knew I was lunchin but I couldn’t even help it, everybody was laughing at me. I didn’t even give a f**k. The police were like “come on man, get down from that pole.” Once I got down, I was strapped in an EMS. While I was in the EMS I told them [paramedics] that I knew magic and that I could get out. They didn’t believe me. [He shows the researcher how he maneuvered his arm and pushed a button that released him from the restraints]. I hopped out of the ambulance and tried to run but the police caught me and beat my a**. Then they took me to PG Hospital. I had been there before. They put me on the psych ward. When my high wore off, I was tripping on them, like why am I in here? Then they let me go.

Aaron reflects back on his first time using SC:

*The first time, I hit the Scooby, I had the Scooby with my man, he lived in my building I only hit it like two times. Man I was tripping. I’m like oh man. It had me feeling like so paranoid like my heart was pumping, my heart beating real fast. So I go to my house, I turn on the shower, I take off all my clothes, the shower on, but I’m just so high I just keep walking around the house, I’m ass naked telling people my job will fire me, my job will fire me.*

Dan echoed Aaron’s sentiments in his description of the effects of SC:

*You get to think you about to die. Man that shit ain’t right. There been plenty of times I thought my heart was about to explode and there ain’t nothing wrong with me. My heart beat is normal, but it feels like you about to go.*

Aaron and Dan’s statements regarding elevated heart rates are commonly reported symptoms among SC users (Harris and Brown 2013).

Study participants reported that the effects of SC use result in erratic behavior. Some of the behaviors described by the participants would be characterized as symptoms of auditory or visual hallucinations, psychosis, or accelerated heart beats. In some cases SC may be used to lower inhibitions with the goal of persuading someone to engage in an act of violence. A distinction is made between the effects felt from natural-marijuana and SC and a contributing factor to the negative side effects experienced from SC use may depend on how they smoke it.

## On papers: criminal justice supervision

The most consistent theme throughout the study was the use of SC among individuals under criminal justice supervision (i.e., probation, parole or electronic monitoring). 82 % of the sample reported on the intersection of criminal justice supervision and SC use. Participants in our sample had disproportionate minority contact with the criminal justice system, 65 % of the sample reported a history of criminal justice involvement. Perrone et al. (2013) found that many users seek out these synthetic cannabinoid substances to avoid civil (loss of employment) and legal (parole or probation revocations) punishments. Furthermore, Perrone et al. (2013) found that most of the users of SC products consumed these products as a substitute for marijuana during drug-testing periods. Participants reported criminal justice supervision as the primary reason why they smoked SC, so as to avoid ‘dirty urine’ in mandatory drug screenings for probation or parole. The street phrase “on papers” was consistently used by participants to capture being under some form of criminal justice supervision. This term was thematic in all of the interviews when participants discussed SC use. Peyton and Vinnie elaborate on how individuals who are under criminal justice supervision smoke SC to avoid criminal sanctions:

*K2 that’s for people who gotta see a PO [probation or parole officer], that’s why that is big, and they want to get high, so they gonna get high.*

*I call it the PO [probation or parole] Pack because everybody using it is on papers.*

Here Eric captures this phenomenon:

Cause these dudes is on papers they smoke it. That s**t don’t show up in the urine.

In some instances we found that individuals released to halfway homes after completing their sentences smoked SC while residing in the halfway home: Russell discusses how SC was the drug of choice among re-entrants in his work release program:

That K2 s**t that’s all that was in there (halfway house), because to be there period you had to take a urine.

In this interview with Kyrie he describes in detail how he was introduced to smoking SC while residing in a halfway house after release from a 10-year prison sentence:

I know a lot of people that smoke it. I call it “K Pack.” Yeah it’s called K-2 or Spice but I call it K Pack. When I was in the halfway house before I was released back home guys in the halfway house use to smoke it because it doesn’t show up in the urine test. I don’t smoke, I just drink, usually I drink Ciroc (a brand of vodka). But dudes in the halfway house were all smoking K Pack. I smoked it once and ended up jumping out of a second floor window after I smoked it. When the people at the halfway house interviewed me about smoking it, I lied and said I was having a panic attack about being out of jail because I had been locked up for so long. But they didn’t believe me because they said this was not the first time someone jumped out of a window or just started lunchin’ (displaying erratic behavior). They were used to that kind of s**t because they had seen it before with people who smoked synthetic weed. So the staff revoked my parole on the grounds that I violated the conditions of my parole and sent me back to jail for a year. I was on the bus going back to jail the next day. That’s how it is in a halfway house people always coming in from prison or going back.

The study participants perceived being “on papers” or criminal justice supervision as a significant contributing factor to the prevalence of SC use in their communities. Study participants reported that when they themselves or their peers have recently engaged in natural-marijuana use and anticipate a planned or random urine analysis that SC is the alternative of choice because it does not get detected in conventional urine analysis tests. Because of its elusive properties, SC is also the drug of choice in work-release programs and half-way houses for those still seeking prohibited euphoric experiences.

## Discussion

SC abuse is a major public health issue. Its use is driven by easy access, low cost, perception as a legal alternative to marijuana use, and the legal/social consequences and criminal sanctions associated with marijuana use, particularly among criminal justice populations. While its use is growing and in some states such as New Hampshire where SC abuse has been declared a state epidemic, more research is needed to inform our understanding of the long-term physiological and pharmacological effects of SC. Previous research on SC use has been limited to examining use among college students (Perrone et al. 2013), white men (Vandrey et al. 2012), and members of the military (Walker et al. 2014). Our findings make contributions to the increasing knowledge on SC use by interviewing Black males under criminal justice supervision about their prevalence of use and the prevalence of use in their community. It’s important to get the perspective of Black males regarding SC given the relationship between SC usage and criminal justice supervision and the disproportionate rates of Black males under criminal justice supervision.

Consistent with previous research conducted on SC, the participants of this study perceived that SC had foreign chemical ingredients that produced negative effects (Vandrey et al. 2012) and that criminal justice supervision was a driving factor behind SC use (Perrone, et al., 2013). Although eighty-eight percent of Vandrey et al. (2012) sample was aware of the harmful chemicals in SC, 11 % believed that SC contained “natural herbs and spices” and 14 % believed that if SC was not safe for human consumption, they would not be marketed and sold in stores. That same neglect is indicated among the participants of this study. Pharmacological knowledge on the short and long-term effects of SC is lacking (Seely et al. 2012). While our study participants expressed concern about the chemical composition of SC none were literate about the intra and inter-batch variability of SC products. This is consistent with previous reports suggesting that SC users are concerned about the health effects of the drug yet have low levels of literacy on the product (CESAR Fax 2013). Furthermore, local public health awareness campaigns on SC do not provide this information. Our findings indicate the importance of educating the public, especially those under criminal justice supervision, on the dangers of consuming SC.

Our findings on the negative short-term effects of SC use are consistent with previous research (Harris and Brown 2013; Seely et al. 2012) as well. Participants in our sample reported experiencing or witnessing someone experience after SC use rapid heartbeat, hallucinations, paranoia, psychosis, irritability, aggressive behavior and withdrawals. These symptoms increase the likelihood of harm and even death (Harris and Brown 2013). This is a cause for concern particularly among vulnerable populations of youth and young adults who are at risk for violent offending and victimization. Effects such as hallucinations, paranoia, psychosis, and aggressive behavior combined with the easy accessibility to firearms for some youth and young adults increases the risk of violent offending and victimization. However, little is known about SC use and firearm-related injury. We found that some participants observed older neighborhood men (old heads) supplying youth with SC to persuade them to engage in violent acts. This is particularly disturbing because it suggests that some violent crimes may be motivated by addiction to SC. More research is needed on the intersection of SC abuse, violent victimization and offending.

The popularity of SC products in low-income neighborhoods is also the result of its availability and low cost. Despite a legal ban on the sale of SC in Prince George’s County and the District of Columbia, stores continue to sell these products making it easily accessible to adolescents and young adults. We also found that individuals are now beginning to produce, package and sell their own SC products. While more research is needed in this area, we can hypothesize that individual producers in the market may increase accessibility and further lower costs. The low cost of SC may also be at the perfect price point for adolescents and young adults who may not be able to afford real marijuana. Our findings indicate that the cost of SC was significantly lower than the cost of marijuana sold on the street. One participant reported buying an ounce of SC for $30. Participants reported that the street price for an ounce of high-grade marijuana was roughly $300 (10 times the price). More research is needed on the marketing, branding, availability and the cost/benefit analysis of SC to determine how these factors influence use and abuse.

## Conclusion

Interpretation of the findings of this study should be considered with caution given the limitations of the study. The data presented was collected from a small sub-sample of 11 participants who participated in a larger pilot study [*N* = 24] on the risk factors for recurrent violent injury among young Black male victims of violent injury. The data cannot and should not be generalized to a broader population of Black males. The findings are limited to Black males between the ages of 18–34 who are under criminal justice supervision and who have experienced a violent injury. Additionally, these findings are limited to a small qualitative sample. Replication of these findings within a larger quantitative data set would provide more empirical support for these qualitative findings. SC abuse has the potential to emerge into a national public health epidemic among adolescents and young adults. Based on our findings and findings from previous studies, we believe this issue warrants serious attention.

The participants of this study perceived that the prevalence of SC use in poor urban neighborhoods was related to the disproportionate number of young Black men who are under criminal justice supervision. Eighty-two percent of the sample reported that the large number of young men under criminal justice supervision contributed to the prevalence of SC use in their communities. Consistent with Perrone et al. (2013) we believe that US criminal justice supervision policies drive the use of SC and the policies provoke the use of SC by many who seek to avoid civil and legal punishments. Furthermore, individuals who have transitioned from marijuana use to SC use to avoid criminal justice sanctions may develop an addiction to SC. More research is needed to determine whether these policies are counter-productive in deterring substance abuse.
